# Expanded Child Tax Credit, Family Health, and Material Hardships

**DOI:** 10.1001/jamanetworkopen.2025.18335

**Published:** 2025-06-30

**Authors:** Stephanie Ettinger de Cuba, Sharon M. Coleman, Allison Bovell-Ammon, Diana Cutts, Megan Sandel, Eduardo Ochoa, Carley Ruemmele, Shailly Gupta Barnes, Charlotte Bruce, Kate Scully, Maureen M. Black, Deborah A. Frank, Félice Lê-Scherban

**Affiliations:** 1 Boston University School of Public Health, Boston, Massachusetts; 2 Boston University Chobanian and Avedisian School of Medicine, Boston, Massachusetts; 3 Boston Medical Center, Boston, Massachusetts; 4 Hennepin Healthcare, Minneapolis, Minnesota; 5 University of Arkansas for Medical Sciences, Little Rock; 6 Kairos Center for Religions, Rights and Social Justice, New York, New York; 7 University of Maryland School of Medicine, Baltimore; 8 RTI International, Research Triangle Park, North Carolina; 9 Drexel University Dornsife School of Public Health, Philadelphia, Pennsylvania

## Abstract

**Importance:**

Understanding associations of cash payments with family health and material hardships can inform national policy design to support families’ economic stability, including an expanded child tax credit (CTC).

**Objective:**

To (1) describe family characteristics, including tax filing and banking status by expanded CTC receipt, and (2) examine longitudinal changes in caregiver and child health and material hardships, including housing stability and food security.

**Design, Setting, and Participants:**

In this longitudinal cohort study, data were drawn from the Children’s HealthWatch COVID Follow-up Study, a study of families surveyed at baseline and 2 waves (January 2018 to March 2020). Primary caregivers of children (≤48 months) participated in English or Spanish in emergency departments or primary care clinics in 4 US cities (Little Rock, Arkansas; Boston, Massachusetts; Minneapolis, Minnesota; and Philadelphia, Pennsylvania). Follow-up surveys were conducted from September 2020 to June 2021 and September 2021 to June 2022. Data were analyzed from March to April 2025.

**Exposure:**

Caregiver-reported CTC receipt.

**Main Outcomes and Measures:**

Caregivers reported whether they had a bank account (ie, were banked), filed taxes, and demographic characteristics. Caregivers also reported child physical health, caregiver physical and mental health, household food insecurity, and housing stability, including rent status (current vs. behind). Adjusted multivariable logistic regression via generalized estimating equations to account for repeated measures was used to assess associations of CTC receipt with health outcomes and hardships over time.

**Results:**

In this cohort of 5813 caregivers (mean [SD] age, 29 [6] years; 1934 non-Latine Black [34.7%]; 2127 Latine [38.3%]; 1275 non-Latine White [22.8%]), 2519 (44.2%) had education beyond high school and 4177 (73.4%) were US born. Of the 879 surveyed after the CTC was implemented, the majority (663 [75.0%]) reported receiving it. Compared with nonrecipient families, a greater proportion of CTC recipients were married or partnered (308 of 663 recipients [47.5%]; 1845 of 5150 nonrecipients [36.6%]), had filed taxes (631 of 662 recipients [95.3%] vs 84 of 194 nonrecipients [43.3%]), were banked (581 of 661 recipients [87.9%] vs 103 of 194 nonrecipients [53.1%]), or were non-Latine White (183 of 663 recipients [28.5%] vs 1092 of 5150 nonrecipients [22.1%]). CTC recipients had significantly lower adjusted odds of hardships, including food insecurity (adjusted odds ratio [aOR], 0.61; 95% CI, 0.47-0.78), housing instability (aOR, 0.64; 95% CI, 0.49-0.83), and being behind on rent (aOR, 0.62; 95%CI 0.48-0.80). Caregivers had lower adjusted odds of anxiety (aOR, 0.67; 95% CI, 0.51-0.89).

**Conclusions and Relevance:**

In this cohort study, CTC recipient families were more likely than nonrecipients to be food secure, stably housed, current on rent, and have caregivers in good mental health, reinforcing associations of increased financial resources with family health and basic needs.

## Key Points

**Question:**

Were the advance monthly payments of the expanded child tax credit (CTC) associated with family health and material hardships during the COVID-19 pandemic?

**Findings:**

In this cohort study among 5813 caregivers with young children who participated in 3 surveys between 2018 and 2022, compared with nonrecipients, CTC recipients had significantly lower adjusted odds of anxiety, experiencing food insecurity, housing instability, and of falling behind on rent.

**Meaning:**

These findings suggest that the expanded CTC supported families’ mental health, food security, and housing stability during a time of increased material hardship in the US.

## Teaser

This cohort study examines associations of health outcomes and material hardships among caregivers with young children with the expanded child tax credit that was issued in response to the COVID-19 pandemic in March 2021.

## Introduction

In response to the COVID-19 pandemic, Congress passed the American Rescue Plan Act in March 2021. The bill made several temporary changes to the child tax credit (CTC), including (1) expanding eligibility for the maximum credit amount to families with low or no income, (2) boosting annual maximum credit per child from $2000 for all children to $3600 for children ages 0 to 6 years and $3000 for children ages 6 to 17 years, and (3) enabling one-half of the annual credit to be paid in monthly installments between July and December 2021 with the balance paid at tax time in spring 2022. These changes made the expanded CTC a near-universal safety-net initiative, with more than 90% of families qualifying for the program. Structural factors like tax filing and whether families had a bank account, as well as communication about the program, were potential barriers to receipt.^1^ Families not required to file taxes may have had more difficulty obtaining the CTC because they had to take proactive action to claim it. In December 2021, Congress failed to extend the American Rescue Plan Act CTC past its expiration date, ending the expanded version of the program.^2,3^

Since the expiration, a growing body of research has demonstrated the expanded CTC was associated with decreased food insecurity, improved dietary quality, decreased child poverty, and decreased housing instability.^4,5,6,7,8,9,10,11,12,13,14^ Compared with traditional annual lump-sum payments, monthly distribution of the CTC reduced income volatility during a time of increased economic hardship.^15^ After 6 months of the expanded program, the national child poverty rate was 5.2%—the lowest recorded rate since the US defined and began tracking poverty in the mid-1960s—attributed in large part to the expanded CTC along with other COVID-19–era benefits.^4^ Poverty, the most well-documented social determinant of health, is associated with multiple negative health outcomes across the lifespan, starting in pregnancy and infancy.^16,17,18,19^

CTC monthly payments were also associated with better physical health and reduced rates of anxiety and depression symptoms among low-income adults with children, particularly non-Latine Black and Latine adults.^10,11,13,14^ These findings complement previous work establishing an association of financial hardship with mental health because caregivers who struggle to afford basic needs experience worse mental health compared with caregivers with higher incomes.^20^ Multiple studies have also demonstrated families used the CTC monthly payments to meet food and housing needs.^5,6,7,9,21^ For example, the expanded CTC has been associated with improved food sufficiency and diet quality.^8,22^ Following the first monthly payment in July 2021, food insufficiency rates among all families with children decreased by 26%^6,7^ and remained lower than pre-expanded CTC levels throughout the remainder of 2021.^2,22^ The expanded CTC has also been associated with better housing stability among families participating in other public benefits.^12^

As interest in interventions that target economic stability grows, further research is needed to more fully understand the association of the expanded CTC with health and well-being, including barriers to receipt and potential to change trajectories of health and hardship over time.^3^ Assessing these associations among families with young children is particularly important because early childhood is a sensitive period of development with the most rapid brain and body growth of their lifetime.^23^

This study, using a unique longitudinal dataset that includes both banking and tax filing, sought to evaluate associations of the expanded CTC with intergenerational health and material hardship outcomes among families with infants and young children. We aimed to understand who did and did not receive the expanded CTC and how it was associated with changing family health and hardship trajectories over time, during a period when material hardship increased dramatically across the US.^24^

## Methods

### Objective and Study Design

This cohort study followed the Strengthening the Reporting of Observational Studies in Epidemiology (STROBE) reporting guideline. The study objectives were to (1) describe baseline and follow-up family characteristics by CTC receipt, including demographics, children’s health insurance, employment, tax filing, and banking status, and (2) examine longitudinal associations of CTC receipt with caregiver mental and physical health and children’s health, as well as household food insecurity and housing instability, including rent status.

The study cohort came from the Children’s HealthWatch COVID Follow-Up Study,^25^ a longitudinal sentinel cohort study of families initially surveyed face-to-face in English or Spanish as part of the Children’s HealthWatch cross-sectional study before the COVID-19 pandemic between January 2018 and March 2020 (baseline) in emergency departments (EDs) or primary care clinics in 4 sites (Boston, Massachusetts; Minneapolis, Minnesota; Philadelphia, Pennsylvania; and Little Rock, Arkansas). Each research site received institutional review board approval annually. Participants were caregivers of young children. Written or verbal informed consent (depending on the institutional review board at each site) at baseline included permission to be recontacted for future study opportunities. Eligibility criteria at baseline at all sites included state residency, child age (≤48 months), English- or Spanish-speaking primary caregiver, living in the child’s household, consenting to be interviewed, and not having been interviewed in the last 6 months. Critically ill or injured children were not approached. Surveys were conducted one-on-one by trained interviewers and included sociodemographics, health measures, household material hardships, and public benefits program participation. Telephone follow-up surveys with previously interviewed caregivers were conducted in wave 1 (September 2020 to June 2021) and wave 2 (September 2021 to June 2022) of the Children’s HealthWatch COVID Follow-Up Study. All caregivers who were surveyed at baseline were invited to participate in wave 1 and also wave 2, regardless of wave 1 participation. Children’s HealthWatch COVID Follow-Up Study surveys included questions about material hardships and program participation since the start of the COVID-19 crisis, including expanded CTC receipt in wave 2. For objective 1 (ie, describe baseline and follow-up family characteristics ), the analytic sample included caregiver and child dyads with complete baseline data. For objective 2 (ie, examining longitudinal associations of CTC receipt with health outcomes), the analytic sample included observations from caregiver and child dyads at baseline, wave 1, and wave 2 (Figure).

**Figure.**
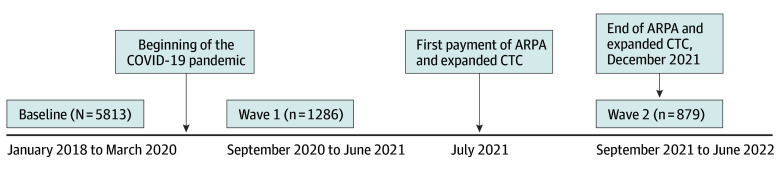
Study Flow Diagram The figure shows the study timeline and number of participants. ARPA indicates American Rescue Plan Act; CTC, child tax credit.

### Outcome Measures

In baseline and follow-up questionnaires, caregivers reported on food security and housing instability. Household food security was assessed using the Six Item Short Form of the Food Security Survey Module.^26,27^ Following standard methods, households were classified as food secure if caregivers reported 0 or 1 affirmative responses and food insecure if caregivers reported 2 or more affirmative responses. Housing instability^28^ was measured as any one or more of the following adverse housing circumstances: children’s lifetime experience of homelessness, multiple moves (≥2 moves in a year), and being behind or current on rent or mortgage (for simplicity, rent status). Rent status was also assessed separately, given the relevance in the COVID-19 pandemic era of falling behind on rent.^28^ For both food insecurity and housing instability, the reference period at baseline was in the past 12 months; in wave 1, since the start of the COVID-19 pandemic (March 2020); and in wave 2, the last 30 days for food insecurity and the last 6 months for housing instability and rent status. Hardship outcomes were used dichotomously and analyzed separately.

At baseline, wave 1, and wave 2, caregivers reported on physical health for themselves and their child (excellent or good vs fair or poor) based on the National Health and Nutrition Examination Survey question.^29^ At wave 1 and wave 2, caregivers reported on their mental health using the Patient Health Questionnaire-4,^30^ a 4-item validated questionnaire asking on how many days in the last 2 weeks the respondent experienced a variety of symptoms, with greater point values assigned to greater symptom frequency. A summed score of 3 or more indicates likely depressive and/or anxiety symptoms.^30^

Caregivers’ race and ethnicity were self-identified and categorized as Black non-Latine, Latine, White non-Latine, or other non-Latine race and ethnicity. Other non-Latine was composed of caregivers identifying multiple races or groups too small in this sample to analyze independently, including Asian, American Indian, or any race or ethnicity not otherwise specified. Race and ethnicity were included to account for known disparities. Maternal nativity was categorized as US-born (including US territories) or immigrant. Public program participation included questions about the Supplemental Nutrition Assistance Program (SNAP), Special Supplemental Nutrition Program for Women, Infants, and Children (WIC), and economic impact payments (ie, stimulus payments). Families were also asked whether they received the expanded CTC (yes or no), had an active checking and/or savings account ( banked vs. unbanked), and had filed taxes for the previous year.

### Statistical Analysis

We used descriptive statistics to examine objective 1 baseline characteristics overall and stratified by CTC receipt. Statistical methods included χ^2^ testing for categorical data and independent samples *t* tests for continuous data. Analysis for objective 2 used multivariable logistic regression models, incorporating time-dependent CTC receipt status as the exposure and time-dependent outcomes, including material hardship and caregiver and child health. To account for repeated measures across baseline and follow-up surveys (wave 1 and wave 2), we fitted the models using generalized estimating equations with an independent working correlation structure. Covariates, chosen a priori, were baseline study site, marital status, caregiver education, caregiver race and ethnicity, maternal nativity, maternal age, child’s health insurance, and child’s age; time-varying covariates were household employment, COVID-19 indicator, stimulus payment receipt, number of children in the household, and months since the baseline interview. For the food insecurity outcome, we also adjusted for SNAP and WIC. Low birth weight (<2500g) was additionally included as a covariate when the child health was the outcome. Data analysis was conducted from March to April 2025 using SAS version 9.4 (SAS Institute). The threshold for statistical significance was a 2-sided *P* < .05.

## Results

Among 5813 caregivers with young children who responded to the baseline survey (mean [SD] age, 29 [6] years; 1934 non-Latine Black [34.7%]; 2127 Latine [38.3%]; 1275 non-Latine White [22.8%]), 1286 responded in wave 1 and 879 responded in wave 2. Of the 5813 caregivers, 2519 (44.2%) had education beyond high school and 4177 (73.4%) were US born. Of the 879 surveyed in wave 2 after the CTC was implemented, the majority reported receipt (663 recipients [75.0%]) . Compared with CTC nonrecipients, a lower proportion of CTC recipients were Latine (215 of 663 recipients [33.5%] vs 1922 of 5150 nonrecipients [38.9%]) and a higher proportion were non-Latine White (183 of 663 recipients [28.5%] vs 1092 of 5150 nonrecipients [22.1%]), and had education beyond high school (372 of 663 recipients [57.1%] vs 2147 of 5150 nonrecipients [42.5%]). CTC recipients compared with nonrecipients also had higher proportions of being married or partnered (308 of 663 recipients [47.5%]; 1845 of 5150 nonrecipients [36.6%]), having private health insurance (96 of 663 recipients [14.8%] vs 505 of 5150 nonrecipients [10.0%]), being banked (581 of 661 recipients [87.9%] vs 103 of 194 nonrecipients [53.1%]), having filed taxes the previous year (631 of 662 recipients [95.3%] vs 84 of 194 nonrecipients [43.3%]), and lower proportions of receiving SNAP (261 of 663 recipients [39.4%] vs 2447 of 5150 nonrecipients [48.0%]) and WIC (208 of 663 recipients [31.4%] vs 2789 of 5150 nonrecipients [54.8%]). Caregiver anxiety and depression and caregiver and child physical health reported as good or excellent were not significantly different between groups. (Table 1)

**Table 1.** Sample Characteristics by Expanded CTC Receipt From the Children’s HealthWatch COVID Follow-Up Study, 2018-2022

Characteristic	Overall participants, No. (%) (N= 5813)	Expanded CTC received, No. (%)		*P* value
		No (n = 5150)^a^	Yes (n = 663)^a^	
CTC receipt among sample interviewed at wave 2 (relevant policy period), No./total No. (%)	879/879 (100)	216/879 (25.0)	663/879 (75.0)	NA
Site				
Boston, Massachusetts	1027 (17.7)	893 (17.3)	134 (20.2)	<.001
Little Rock, Arkansas	2313 (39.8)	1974 (38.3)	339 (51.1)	
Minneapolis, Minnesota	730 (12.6)	674 (13.1)	56 (8.4)	
Philadelphia, Pennsylvania	1743 (30.0)	1609 (31.2)	134 (20.2)	
Caregiver characteristics				
Education				<.001
Never, elementary, or some high school	996 (17.5)	916 (18.1)	80 (12.3)	
High school or GED	2189 (38.4)	1990 (39.4)	199 (30.6)	
Technical school, college graduate, master’s, or above	2519 (44.2)	2147 (42.5)	372 (57.1)	
Marital status				
Single	2290 (40.2)	2079 (41.2)	211 (32.5)	<.001
Married, partnered, cohabiting	2153 (37.8)	1845 (36.6)	308 (47.5)	
Separated, divorced, widowed	1247 (21.9)	1117 (22.2)	130 (20.0)	
Maternal race and ethnicity				
Black, non-Latine	1934 (34.7)	1722 (34.9)	212 (33.0)	
Latine	2137 (38.3)	1922 (38.9)	215 (33.5)	
White, non-Latine	1275 (22.8)	1092 (22.1)	183 (28.5)	
Other, non-Latine^a^	235 (4.2)	203 (4.1)	32 (5.0)	
Maternal nativity				
US-born	4177 (73.4)	3707 (73.5)	470 (72.6)	.67
Immigrant	1516 (26.6)	1339 (26.5)	177 (27.4)	
Maternal age at baseline, mean (SD), y	29 (6)	29 (6)	30 (6)	<.001
Child characteristics				
Child health insurance				
Public	4954 (86.6)	4414 (87.0)	540 (83.2)	<.001
No insurance	165 (2.9)	152 (3.0)	13 (2.0)	
Private	601 (10.5)	505 (10.0)	96 (14.8)	
Low birth weight (<2500g)	801 (14.5)	708 (14.5)	93 (14.7)	.86
Child age at baseline, mean (SD), mo	20.32 (13.84)	20.30 (13.85)	20.54 (13.76)	.63
Time-varying characteristics				
≥1 person employed in household	4814 (83.5)	4266 (83.6)	548 (82.8)	.62
Time since baseline survey, mean (SD), mo	8.40 (14.70)	4.80 (10.90)	36.80 (8.20)	<.001
No. of children 0-18 mo in household, mean (SD)	2 (1)	2 (1)	3 (1)	<.001
SNAP participation	2708 (47.0)	2447 (48.0)	261 (39.4)	.62
WIC participation	2997 (52.1)	2789 (54.8)	208 (31.4)	<.001
Stimulus check receipt	1219 (21.0)	654 (12.7)	565 (86.8)	<.001
Caregiver anxiety	345 (21.9)	205 (22.4)	140 (21.1)	.55
Caregiver depression	200 (12.7)	123 (13.5)	77 (11.6)	.27
Caregiver physical health				
Good or excellent	4519 (78.5)	4018 (78.8)	501 (75.6)	.05
Fair or poor	1240 (21.5)	1078 (21.2)	162 (24.4)	
Child physical health				
Good or excellent	5290 (91.8)	4690 (92.0)	600 (90.5)	.19
Fair or poor	471 (8.2)	408 (8.0)	63 (9.5)	
Wave 2 CTC-related characteristics, No./total No. (%)				
Banked (active savings or checking account)	684/855 (80.0)	103/194 (53.1)	581/661 (87.9)	<.001
Filed taxes	715/856 (83.5)	84/194 (43.3)	631/662 (95.3)	<.001

Abbreviations: CTC, child tax credit; GED, general educational development; SNAP, Supplemental Nutrition Assistance Program; WIC, Special Supplemental Nutrition Program for Women, Infants and Children.

^a^ Maternal other, non-Latine race and ethnicity includes caregivers identifying multiple races or groups too small in this sample to analyze independently, including Asian, American Indian, or any race or ethnicity not otherwise specified.

CTC recipients had significantly lower odds of caregiver anxiety (adjusted odds ratio [aOR], 0.67; 95% CI, 0.51-0.89), household food insecurity (aOR, 0.61; 95% CI, 0.47-0.78), housing instability (aOR, 0.64; 95% CI, 0.49-0.83) and, specifically among unstable housing conditions, lower odds of being behind on rent or mortgage (aOR, 0.62; 95% CI, 0.48-0.80) compared with nonrecipients (Table 2). No significant differences were observed in child or caregiver physical health, or caregiver depression.

**Table 2.** Adjusted Child, Parent, and Household Outcomes by CTC Receipt

Outcome	CTC, aOR (95%CI)
Child good or excellent health	0.74 (0.49-1.11)
Caregiver good or excellent health	0.96 (0.74-1.25)
Caregiver depression^a^	0.83 (0.59-1.18)
Caregiver anxiety^a^	0.67 (0.51-0.89)^b^
Household food insecurity	0.61 (0.47-0.78)^b^
Rent or mortgage status (behind on rent or mortgage)	0.62 (0.48-0.80)^b^
Housing instability	0.64 (0.49-0.83)^b^

Abbreviations: aOR, adjusted odds ratio; CTC, child tax credit.

^a^ These models used follow-up data only because the Patient Health Questionnaire-4 was not available at baseline. The reference group for all outcomes is no CTC. Covariates included baseline characteristics (study site, marital status, caregiver education, caregiver race and ethnicity, maternal nativity, maternal age, child’s health insurance status, and child’s age) and time-varying characteristics (employment in household, COVID-19 indicator, stimulus payment, number of children in the household, and months since the baseline interview). The food insecurity model additionally controlled for participation in the Supplemental Nutrition Assistance Program and Special Supplemental Nutrition Program for Women, Infants and Children. The child health outcome additionally controlled for low birth weight.

^b^ *P* value <.05.

## Discussion

Economic stability is vital for optimal parent and child health and development.^3,23^ Results from this cohort study demonstrate expanded CTC receipt was associated with less anxiety among caregivers and improved household food security and stable housing, including greater likelihood of catching up on rent after being behind early in the COVID-19 pandemic. When families can afford necessary expenses, including food, rent, utilities, childcare, and health care without sacrificing other needs, everyone in the household experiences better health outcomes.^28,31,32,33^ Health impacts are interrelated and often chronic, such that longer experiences of poverty-related hardships in childhood can increase the risk of poor health and associated negative outcomes throughout childhood and adulthood.^34,35,36,37^

In our unique sample of families with young children, better caregiver mental health could have ripple effects by reducing the number of health challenges facing the whole family.^38,39^ In addition to the standalone benefits of food security and better housing stability (especially being up-to-date on rent), security and stability can decrease the health risks of other material hardships. Housing instability and food insecurity are interrelated, and both are associated with worse child and caregiver health, including child developmental delays and hospitalizations.^28,40^ This study aligns with others examining caregivers’ health and family economic stability. In a study of SNAP-participant families with children younger than 18 years, monthly CTC payments were associated with reductions in both the amount owed and rate of overdue payments, as well as fewer people residing in their household, likely decreasing household crowding, which is associated with children’s less optimal school-readiness.^12,41^

Despite positive outcomes from the expanded CTC, many eligible families in our sample did not receive it, consistent with other studies.^5,42,43,44^ All of the families in our overwhelmingly low-income dataset would have been eligible based on income, but several factors may contribute to this gap. For example, to receive the fully refundable expanded CTC, individuals had to file a tax return or submit information to the Internal Revenue Service (IRS). Those who do not routinely file taxes were eligible but had to submit information through the IRS nonfiler portal.^45^ Previous research has demonstrated families who do not routinely file tax returns experience barriers that can prevent them from accessing benefits, such as not having a bank account or a consistent home address to receive the payments.^42,46,47^ Tax filing and banking status could be viewed both as a potential confounder and as a proxy for financial access. In our context, they are highly concordant (80% for banking and 87% for tax filing) with CTC receipt status and likely act as partial mediators (eg, CTC disbursement via direct deposit requires an account) rather than a pure confounder. Adjusting for such variables can induce overadjustment bias by artificially blocking part of the policy’s intended effect. All of the families in our overwhelmingly low-income dataset should have been eligible based on income.^48^

Eligibility for immigrant families was also confusing. Families that were eligible to file taxes with an individual taxpayer identification number (ITIN) had to first apply for an ITIN if they did not already have one. However, IRS backlogs were substantial, slowing ITIN processing and immigrant families’ ability to file for the CTC. In addition, many feared enrolling in the CTC would constitute a public charge, putting their immigration status at risk.^42,49,50^ Logistical barriers combined with misconceptions resulted in fewer eligible immigrant families receiving the monthly payments than US-born families, a finding aligned with national trends.^42,45,51^

Finally, others have demonstrated that deep-rooted distrust in the government among poor, historically marginalized communities has contributed to skepticism and misconceptions about the CTC.^52^ These ranged from misconceptions that the monthly payments would need to be repaid or that one had to have paid employment to receive the CTC, both of which were untrue.^53,54^ Families may feel skeptical of programs that seem to promise free money, which can feel too good to be true or difficult to distinguish from scams.^46^ While government agencies and many community-based organizations engaged in outreach efforts about the expanded CTC, high levels of CTC nonreceipt, paired with general distrust about program duration and requirements, underscore the ways tailored communication could have improved participation^55,56,57^ This, too, may help explain the gap between CTC eligibility and participation.

Following the expanded CTC’s expiration, child poverty rates more than doubled^58^ and food insufficiency returned to pre-expansion levels.^24,59,60,61^ With termination of other COVID-19 pandemic–era resources, an expanded, inclusive, and monthly CTC is needed for families to keep up with rising costs of living,^62^ particularly with no increase in the national minimum wage over more than 15 years.^63,64,65^ Permanently implementing an expanded CTC could change health and well-being trajectories for many families currently experiencing material hardship, improving both health and economic stability among families in the US.^3,7,12,21,66,67^

Several tax cuts from the first Trump administration are set to expire in 2025, setting the stage for debate and legislative action on tax policy.^68^ This is an opportunity for health professionals to bring attention back to the robust evidence of the expanded CTC’s positive implications for families with young children,^3^ ensuring a future expanded CTC includes both families without a minimum income requirement and those with immigrant family members, as well as support for the IRS to operate independently and efficiently and for robust community outreach.^42^ The 2025 debate could provide the opportunity to permanently expand the CTC, in turn reducing child poverty, improving food security, and advancing stable housing and good health for families. Considering disproportionate hardships experienced by racially and ethnically minoritized individuals during the COVID-19 pandemic,^24^ in addition to well-documented associations of health with structural racism, future work should explore the expanded CTC’s impact from a racial equity lens and develop strategies to reduce structural barriers experienced by systemically marginalized families.^42,69^

### Strengths and Limitations

This study’s strengths include its multisite dataset design with widely diverse participant race, ethnicity, and nativity. The study provides data on an underrepresented group of families with young children that is both difficult to reach through online questionnaires (given generally unreliable internet access) and highly vulnerable to economic hardship.

This study also has limitations. The longitudinal sample was initially enrolled at baseline in the cross-sectional Children’s HealthWatch sentinel study. Potential for sample selection bias exists because participants were caregivers of young children seeking health care in EDs or primary care clinics, potentially limiting findings’ generalizability. Children identified in EDs may be more vulnerable to negative effects of CTC nonreceipt; their inclusion may bias health outcomes away from the null. The prospective design of the longitudinal sample allows for understanding associations over time; however, differential follow-up may bias results. The sample is not nationally representative, although it was drawn from a sentinel study design intended to monitor a population at highest risk of showing early signs of either benefit or harm from policy-related exposures. The sample was similar in family demographics and child health outcomes to low-income families in the National Survey of Children’s Health.^70^ All hardships, program participation, and other data were caregiver-reported and thus subject to recall bias, although the food security measure was validated and the measure of housing instability has been used in multiple studies.^28,40^ Families with barriers to CTC receipt, including bank status and nativity, may also have barriers to public assistance participation and thus fewer financial resources; this could contribute to greater financial hardship among CTC nonrecipients and, in turn, worse caregiver and child health outcomes. Sample size precluded us from evaluating potential effect heterogeneity, such as those documented in prior studies of the CTC.

## Conclusions

In this cohort study of caregivers with young children, expanded CTC recipients compared with nonrecipients were less likely to have anxiety, and families were more likely to be food secure, stably housed, and current on rent. Despite the substantial strides made by the expanded CTC, expiration reversed many of its gains. To maximize the potential benefits of a permanently expanded CTC, further research is needed on enrollment barriers, particularly among immigrant families and families from systemically marginalized communities. As policymakers consider future tax reforms, this study can be used to support efforts to reinstate the expanded CTC for better health and economic stability for all families and children.

## Notes

Supplement.
Data Sharing Statement
