# Supplementary material for: Expanded Child Tax Credit, Family Health, and Material Hardships
Source: JAMA Netw Open. 2025 Jun 30;8(6):e2518335. doi: 10.1001/jamanetworkopen.2025.18335 (PMC12210083; doi:10.1001/jamanetworkopen.2025.18335)
Supplement: Supplement. — Data Sharing Statement [file jamanetwopen-e2518335-s001.pdf]

Data Sharing Statement

de Cuba. Expanded Child Tax Credit, Family Health, and Material Hardships. *JAMA Netw Open*. Published June 30, 2025. doi:10.1001/jamanetworkopen.2025.18335

Data
Data available: No
